# Fatal Chronic Varicella‐Zoster Viral Infection in a Young Man With Chediak–Higashi Syndrome

**DOI:** 10.1111/pde.70082

**Published:** 2025-11-20

**Authors:** Albane Badet, Clément Pruvot, Zoé Manssens, Wadih Abou‐Chahla, Sébastien Buche

**Affiliations:** ^1^ Department of Dermatology Lille University Hospital Lille France; ^2^ University of Lille Lille France; ^3^ Pediatric Dermatology Department Lille University Hospital Lille France; ^4^ Pathology Department Lille University Hospital Lille France; ^5^ Pediatric Hematology and Oncology Department, Jeanne de Flandre Hospital Lille University Hospital Lille France

**Keywords:** acyclovir resistance, Chediak–Higashi syndrome, chronic VZV infection, herpesviruses, immunodeficiency, JAK inhibitors, macrophage activation syndrome

## Abstract

Chediak–Higashi syndrome (CHS) is a rare autosomal recessive primary immunodeficiency characterized by partial oculocutaneous albinism, neurologic involvement, and a predisposition to severe infections. Patients are particularly susceptible to developing hemophagocytic lymphohistiocytosis (HLH), which significantly worsens prognosis. We report the case of a 19‐year‐old male with CHS, under long‐term immunosuppressive therapy for chronic HLH, who developed chronic VZV infection. Despite treatment with valacyclovir, the lesions progressed, and virological investigations confirmed the diagnosis of acyclovir‐resistant varicella‐zoster virus (VZV). The clinical course was marked by the appearance of neurological symptoms and a fatal outcome. To our knowledge, this is the first reported fatal case of chronic VZV infection with systemic dissemination in a patient with CHS. This case highlights the risk of resistance associated with long‐term prophylaxis and the complexity of managing herpesvirus infections in immunocompromised patients receiving JAK inhibitors.

## 1 Introduction

Chediak–Higashi syndrome (CHS) is a rare autosomal recessive disorder of unknown prevalence, combining partial oculocutaneous albinism, various neurological manifestations, innate and adaptive immune deficiency. It is caused by mutations in the *LYST* gene (lysosomal trafficking regulator), leading to intracellular lysosomal transport dysfunction and resulting in defective immune cell function. Patients are particularly vulnerable to severe infections [1]. CHS belongs to the group of diseases associated with partial oculocutaneous albinism and immunodeficiency (OCA‐ID) [2]. The prognosis is poor, with death usually occurring between ages 20 and 40, due to bacterial infections and complications of hemophagocytic lymphohistiocytosis (HLH), which occurs in 50% to 80% of cases [1, 3, 4]. Partial albinism manifests with sometimes subtle skin hypopigmentation, light hair with a characteristic silvery sheen, and ocular signs such as iris and retinal hypopigmentation, nystagmus, and reduced visual acuity. Neurological manifestations are frequent and variable, ranging from early cognitive disorders to progressive neurodegeneration, including cerebellar ataxia and sensorimotor neuropathy [2, 5]. We report a case of chronic acyclovir‐resistant varicella‐zoster virus (VZV) infection with a fatal outcome in a patient with CHS, treated with immunosuppressive therapy for macrophage activation syndrome.

## 2 Case Report

A 19‐year‐old male with CHS syndrome complicated by neurobehavioral disorders and chronic hemophagocytic lymphohistiocytosis (HLH) was treated with ruxolitinib 20 mg twice daily, weekly subcutaneous immunoglobulins, and prednisone 2.5 mg twice daily. He was hospitalized in Month 0 for primary varicella infection. Intravenous acyclovir (500 mg/m^2^ every 8 h for 5 days) was initiated, with a favorable clinical course. He was discharged with oral valacyclovir (1 g three times daily for 10 days). In Month 1, he developed right thoracic herpes zoster, which responded to valacyclovir (1 g three times daily for 7 days). Two weeks later, a contralateral recurrence occurred, evolving into chronic lesions. Valacyclovir was maintained at 500 mg twice daily.

**FIGURE 1:**
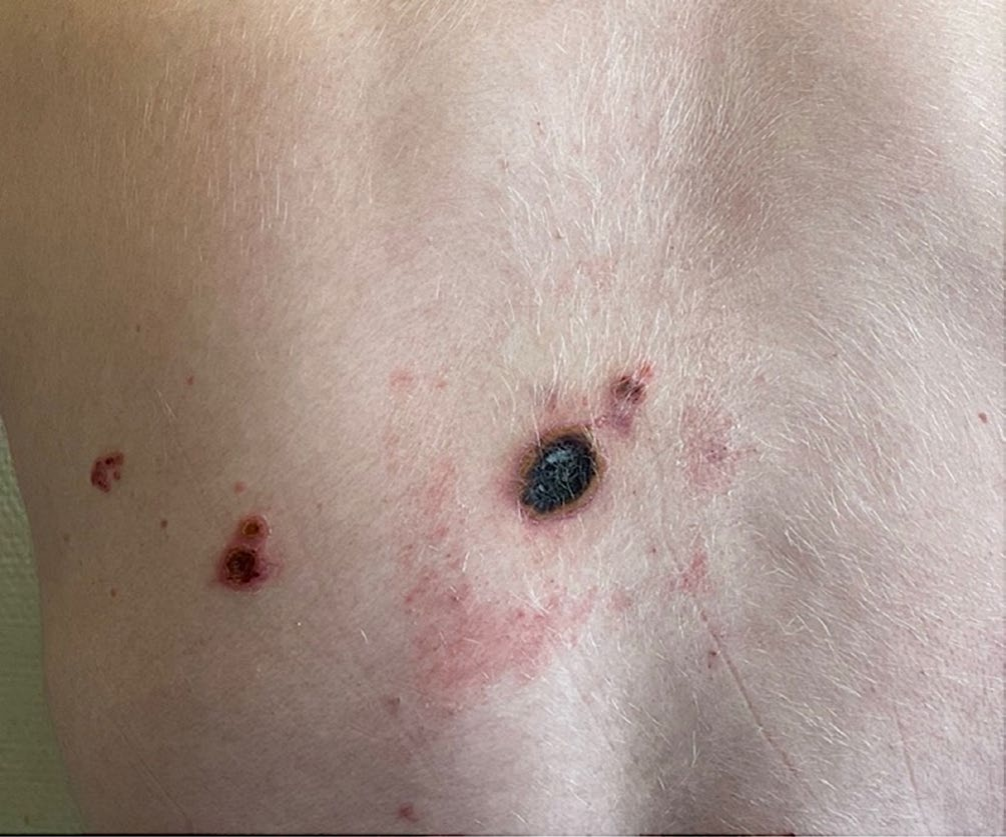
M+6 Clinical presentation. Necrotic interscapular lesion measuring 2 cm in greatest diameter.

**FIGURE 2:**
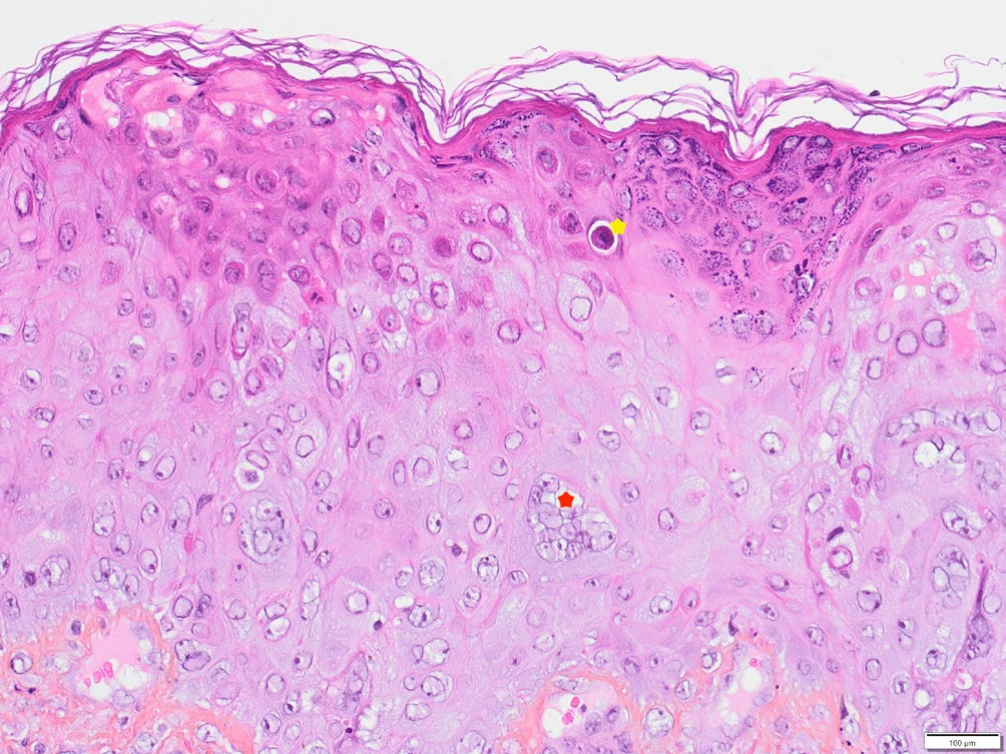
Dorsal Skin biopsy, HES coloration, ×200: Keratinocyte necrosis (yellow star), multiple viral inclusion bodies, including occasional muriform cells (red star).

In month 6, a 4‐cm ulcerated lesion appeared on the left scapula with slightly papular, crusted borders and polymorphic vesicular lesions of various ages (Figure 1). A skin biopsy (Figure 2) revealed mononuclear lymphocytic infiltrate, keratinocyte necrosis, and viral inclusions with multinucleated giant cells, suggestive of herpesvirus infection. Immunohistochemistry for herpes simplex virus (HSV) was negative; polymerase chain reaction (PCR) for varicella‐zoster virus (VZV) on the biopsy was positive, confirming chronic VZV infection. Bacterial cultures from the biopsy were negative.

**FIGURE 3:**
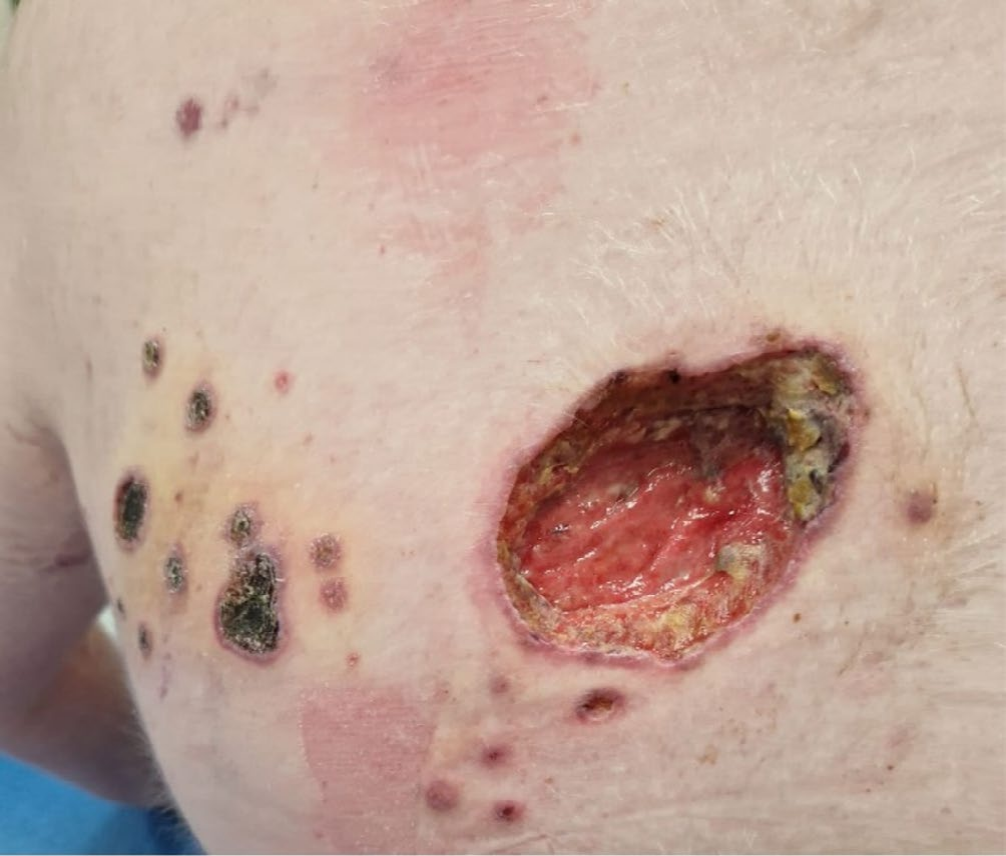
M+7. Appearance of new lesions and deepening of the interscapular ulcer.

Valacyclovir was increased to 1 g three times daily, but the scapular lesion worsened, becoming deep and necrotic (Figure 3). In Month 8, a second biopsy for antiviral resistance genotyping identified a mutation in the VZV thymidine kinase gene, confirming resistance to acyclovir. Compassionate use of amenamevir (200 mg twice daily) was authorized. Due to persistent HLH, reduction of immunosuppressive therapy was not possible.

**FIGURE 4:**
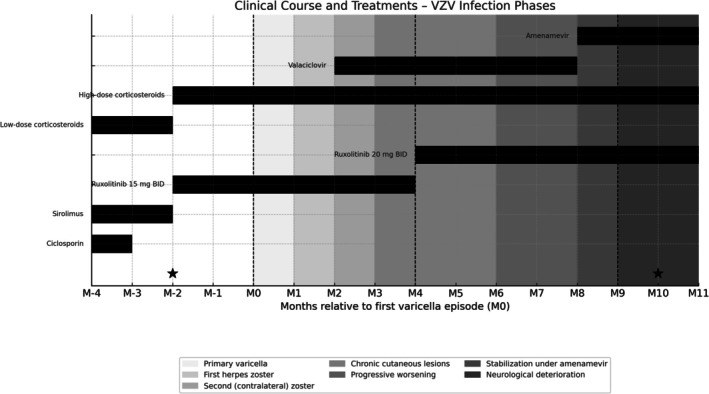
Clinical course and treatments—VZV infection phases. This figure summarizes the patient's clinical course from the onset of varicella (M0) through subsequent VZV reactivations and complications. Grayscale background bands illustrate the different phases of VZV infection: Primary varicella, first and second herpes zoster episodes, chronic skin lesions, progressive worsening, stabilization under amenamevir, and neurological deterioration. Black horizontal bars represent the immunosuppressive and antiviral treatments administered over time. Low‐dose corticosteroids were maintained from M–4 to M–2, then replaced by high‐dose corticosteroids from M–2 onward. Ruxolitinib was introduced at M–2 alongside the withdrawal of sirolimus. Major clinical events are marked with dashed vertical lines (M0, M4, M9). HLH flare‐ups are indicated with black stars (★) at M–2 and M+10.

In month 10, the patient presented with generalized tonic–clonic seizures requiring intensive care unit admission. VZV PCR from blood was positive; HSV 1 and 2 were negative. Brain magnetic resonance imaging (MRI) revealed meningoencephalitis with Fluid‐Attenuated Inversion Recovery (FLAIR) hyperintensities in the left frontotemporal region and infratentorial areas, along with leptomeningeal enhancement, suggestive of VZV infection, though neuromeningeal HLH involvement could not be excluded. Lumbar puncture was attempted three times unsuccessfully (Figure 4). Despite empirical antibiotic therapy (cefotaxime 300 mg/kg/day, amoxicillin 2 g every 6 h) and intravenous foscarnet (120 mg/kg/day), the patient deteriorated and died.

## 3 Discussion

Our patient developed a fatal case of chronic VZV infection in the context of severe immunosuppression, resulting from both an innate and adaptive immune defect and long‐term treatment for chronic HLH. Although HSCT can correct the hematologic and immune defects of CHS, it does not halt the progression of established neurological disease [6]. In this patient, severe and irreversible neurological sequelae, present since early childhood, contraindicated HSCT, and long‐term immunosuppressive therapy was therefore maintained to control the chronic HLH. Chronic VZV infection is rare and usually occurs in patients with acquired immunodeficiency, such as HIV infection, and less commonly in solid organ transplant recipients or those with hematologic malignancies. Lesions are typically crusted or hyperkeratotic, and occasionally ulcerated, with a nonspecific distribution. Only one case of dermatomal distribution has been reported [7]. To date, no cases of fatal chronic cutaneous VZV infection with visceral involvement have been reported in the literature [7].

Prevention relies on vaccination, either of the at‐risk patient or their close contacts. At the time of this case, only live‐attenuated vaccines were available, which were contraindicated in our immunosuppressed patient. The recombinant subunit vaccine SHINGRIX is now approved for immunocompromised individuals over the age of 18.

First‐line treatment of varicella relies on acyclovir or valacyclovir, but resistance is increasingly reported in immunocompromised patients [8]. Resistance typically results from mutations in the viral thymidine kinase gene or, less frequently, the DNA polymerase gene. Acyclovir requires phosphorylation by viral thymidine kinase to become active. Mutations in this enzyme prevent activation, leading to therapeutic failure.

Acyclovir and valacyclovir resistance primarily affects immunocompromised patients, with reported prevalence around 5% in solid organ transplant recipients and up to 30% in stem cell transplant recipients. Prolonged antiviral exposure, especially at subtherapeutic doses, contributes to resistance [9]. In cases of strong clinical suspicion of VZV infection that do not respond to appropriate antiviral therapy, acyclovir resistance should be considered and investigated. Amenamevir is an antiviral agent with a mechanism of action distinct from that of acyclovir, as it does not require phosphorylation by thymidine kinase (TK). It acts by inhibiting the helicase‐primase complex. It is used as an alternative in cases of suspected or confirmed acyclovir resistance. Amenamevir, initially chosen in our patient due to its oral formulation, is not approved for the treatment of VZV‐related central nervous system complications. Its cerebrospinal fluid (CSF) bioavailability is only 10%, compared to 50% for acyclovir. This limited central nervous system penetration reduces its efficacy against central nervous system infections and may explain the development of probable varicella encephalitis in our patient [10].

HLH is a rare (1 in 8 million) and life‐threatening condition, with reported mortality ranging from 20% to 80% depending on the study. It is characterized by systemic hyperinflammation caused by an excessive but ineffective activation of the immune system, leading to multiorgan failure. HLH may be genetic or acquired, triggered by infection, autoinflammatory disease, malignancy, or immunodeficiency. Diagnosis requires at least five of the following eight criteria: persistent fever, splenomegaly, cytopenias, hypertriglyceridemia or hypofibrinogenemia, hyperferritinemia, decreased NK cell activity, elevated sCD25 levels, or documented hemophagocytosis.

Neurological involvement in HLH is frequent, particularly in children. It may range from peripheral neuropathies and focal deficits to encephalopathy, seizures, or even coma. CSF analysis may reveal pleocytosis, while brain MRI often shows nonspecific abnormalities such as leptomeningeal enhancement, white matter lesions, or signs of ischemia [10].

In our case, the neurological findings could not definitively distinguish VZV‐related meningoencephalitis from neuromeningeal HLH. Treatment of HLH is based on corticosteroids and immunosuppressants such as cyclosporin, etoposide, hematopoietic stem cell transplant, or more recently JAK inhibitors [10, 11]. In our patient, the chronic nature of HLH made it impossible to reduce immunosuppressive therapy, thereby complicating the management of the chronic infection and perpetuating a vicious cycle between HLH exacerbation and viral persistence. The use of a Janus kinase (JAK) inhibitor to control HLH may have influenced the course of the infection. JAK1 and JAK2 inhibitors (ruxolitinib, tofacitinib), by inhibiting interferon (IFN), tumor necrosis factor (TNF), and IL‐12 signaling, interfere with the Th1 response and the production of VZV‐specific T cells (Table 1). Tofacitinib reduces IFN‐γ production and significantly decreases the proliferation, activation, and CXCR3 expression of VZV‐specific CD4+ T cells in a dose‐dependent manner, potentially impairing Th1‐mediated control of latent VZV infection [12].

**TABLE 1:** HLH biomarker trends and clinical activity summary.

Month	Ferritin	Hemoglobin	Platelets	Neutrophils	CRP	Fever	HLH activity
M‐2	↑	↓↓	↓↓	↓↓	↔	N	High
M‐1	↑	↓↓	↓↓	↓	↔	N	High
M0	↑	↑	↑	↓	↔	Y	Partial
M2	—	↔	↓	↓	↔	Y	Moderate
M3	—	↔	↓	↓	↔	Y	Moderate
M4	↔	↔	↔	↓	↔	Y	Stable
M6	—	↓	↓	↓	↔	Y	Moderate
M8	↑↑↑	↓↓	↓↓	↓↓	↑	Y	High
M10	—	↓	↓	↓	↑↑	Y	Severe

A twofold increase in the incidence of herpes zoster has been reported in patients with rheumatoid arthritis treated with tofacitinib compared to those receiving biologic agents such as adalimumab, certolizumab, etanercept, abatacept, rituximab, or tocilizumab [13]. Similar trends have been observed for other JAK inhibitors [14].

## 4 Conclusion

Our case highlights the clinical challenges posed by chronic, drug‐resistant viral infections in the context of immunosuppressive therapy. Although rare, chronic acyclovir‐resistant VZV infection represents a potentially life‐threatening complication. Prolonged use of prophylactic antivirals in immunocompromised patients promotes the emergence of resistance, compromising the effectiveness of standard therapies and limiting treatment options. Moreover, the use of JAK inhibitors, while effective in controlling HLH, may impair antiviral immune responses, particularly against varicella‐zoster virus, thereby increasing the risk of viral reactivations and chronic infections.

This case underscores the importance of heightened vigilance for antiviral resistance and the need for close monitoring of infections in immunocompromised patients, especially those receiving JAK inhibitors. The use of third‐line antivirals such as amenamevir, though promising, has limitations in terms of central nervous system bioavailability, as illustrated by the unfavorable outcome observed in our patient.

## Ethics Statement

Written informed consent for publication could not be obtained from the patient due to his death; however, all potentially identifying information has been anonymized in accordance with the journal's policy.

## Conflicts of Interest

The authors declare no conflicts of interest.

## Data Availability

The data that support the findings of this study are available on request from the corresponding author. The data are not publicly available due to privacy or ethical restrictions.

## References

[1] J. Kaplan , I. De Domenico , and D. M. Ward , “Chediak‐Higashi Syndrome,” Current Opinion in Hematology 15, no. 1 (2008): 22–29. 18043242 10.1097/MOH.0b013e3282f2bcce

[2] L. Dotta , S. Parolini , A. Prandini , et al., “Clinical, Laboratory and Molecular Signs of Immunodeficiency in Patients With Partial Oculo‐Cutaneous Albinism,” Orphanet Journal of Rare Diseases 8 (2013): 168. 24134793 10.1186/1750-1172-8-168 PMC3856608

[3] M. L. Lozano , J. Rivera , I. Sánchez‐Guiu , and V. Vicente , “Towards the Targeted Management of Chediak‐Higashi Syndrome,” Orphanet Journal of Rare Diseases 9 (2014): 132. 25129365 10.1186/s13023-014-0132-6 PMC4243965

[4] I. Maaloul , J. Talmoudi , I. Chabchoub , et al., “Chediak–Higashi Syndrome Presenting in Accelerated Phase: A Case Report and Literature Review,” Hematology/Oncology and Stem Cell Therapy 9, no. 2 (2016): 71–75. 26254864 10.1016/j.hemonc.2015.07.002

[5] C. Raghuveer , S. C. Murthy , M. N. Mithuna , and T. Suresh , “Silvery Hair With Speckled Dyspigmentation: Chediak‐Higashi Syndrome in Three Indian Siblings,” International Journal of Trichology 7, no. 3 (2015): 133–135. 26622160 10.4103/0974-7753.167462 PMC4639960

[6] M. L. Talbert , M. C. V. Malicdan , and W. J. Introne , “Chediak‐Higashi Syndrome,” Current Opinion in Hematology 30, no. 4 (2023): 144–151. 37254856 10.1097/MOH.0000000000000766 PMC10501739

[7] O. Wauters , E. Lebas , and A. F. Nikkels , “Chronic Mucocutaneous Herpes Simplex Virus and Varicella Zoster Virus Infections,” Journal of the American Academy of Dermatology 66, no. 6 (2012): e217–e227. 21056516 10.1016/j.jaad.2010.07.011

[8] H. H. Schalkwijk , R. Snoeck , and G. Andrei , “Acyclovir Resistance in Herpes Simplex Viruses: Prevalence and Therapeutic Alternatives,” Biochemical Pharmacology 206 (2022): 115322. 36309081 10.1016/j.bcp.2022.115322

[9] S. Tada , Y. Kaito , A. Watanabe , et al., “Varicella‐Zoster Meningitis and Myelitis After Herpes Zoster Dermatitis Treatment With Amenamevir: A Case Series and Literature Review,” Cureus 16, no. 2 (2024): e54775. 38524092 10.7759/cureus.54775 PMC10961168

[10] A. Hayden , S. Park , D. Giustini , A. Y. Y. Lee , and L. Y. C. Chen , “Hemophagocytic Syndromes (HPSs) Including Hemophagocytic Lymphohistiocytosis (HLH) in Adults: A Systematic Scoping Review,” Blood Reviews 30, no. 6 (2016): 411–420. 27238576 10.1016/j.blre.2016.05.001

[11] C. Keenan , K. E. Nichols , and S. Albeituni , “Use of the JAK Inhibitor Ruxolitinib in the Treatment of Hemophagocytic Lymphohistiocytosis,” Frontiers in Immunology 12 (2021): 614704. 33664745 10.3389/fimmu.2021.614704 PMC7923355

[12] G. Almanzar , F. Kienle , M. Schmalzing , A. Maas , H. P. Tony , and M. Prelog , “Tofacitinib Modulates the VZV‐Specific CD4+ T Cell Immune Response in Vitro in Lymphocytes of Patients With Rheumatoid Arthritis,” Rheumatology (Oxford, England) 58, no. 11 (2019): 2051–2060. 31106368 10.1093/rheumatology/kez175

[13] J. R. Curtis , F. Xie , H. Yun , S. Bernatsky , and K. L. Winthrop , “Real‐World Comparative Risks of Herpes Virus Infections in Tofacitinib and Biologic‐Treated Rheumatoid Arthritis Patients,” Annals of the Rheumatic Diseases 75, no. 10 (2016): 1843–1847. 27113415 10.1136/annrheumdis-2016-209131 PMC5553444

[14] F. Sunzini , I. McInnes , and S. Siebert , “JAK Inhibitors and Infections Risk: Focus on Herpes Zoster,” Therapeutic Advances in Musculoskeletal Disease 12 (2020): 1759720X20936059. 10.1177/1759720X20936059 PMC7328488 32655703

